# Effectiveness of a fourth dose of mRNA COVID-19 vaccine against all-cause mortality in long-term care facility residents and in the oldest old: A nationwide, retrospective cohort study in Sweden

**DOI:** 10.1016/j.lanepe.2022.100466

**Published:** 2022-07-13

**Authors:** Peter Nordström, Marcel Ballin, Anna Nordström

**Affiliations:** a Department of Community Medicine and Rehabilitation, Unit of Geriatric Medicine, Umeå University, Umeå, Sweden; b Department of Public Health and Clinical Medicine, Section of Sustainable Health, Umeå University, Umeå, Sweden; c School of Sport Sciences, UiT the Arctic University of Norway, Tromsø, Norway

**Keywords:** COVID-19, Nursing home residents, Vaccination

## Abstract

**Background:**

The effect of a fourth dose of COVID-19 vaccine on the risk of death in the oldest and frailest individuals is unknown.

**Methods:**

Two matched cohorts were formed using Swedish nationwide registers. In the first, residents of long-term care facilities (LTCFs) given a fourth dose of an mRNA vaccine from 1 January 2022 onwards were matched 1:1 on birth year and county of residence to residents given at least a third dose (*N* = 24,524). In the second, all individuals aged ≥80 years given a fourth dose were matched 1:1 to individuals given at least a third dose (*N* = 394,104). Cox regression models were used to estimate hazard ratios for all-cause mortality in fourth-dose recipients as compared with in third-dose recipients, with relative vaccine effectiveness (VE) estimated as 1 minus the hazard ratio.

**Findings:**

From 7 days after baseline and onwards, there were 1119 deaths in the LTCF cohort during a median follow-up of 77 days and a maximum follow-up of 126 days. During days 7 to 60, the VE of the fourth dose was 39% (95% CI, 29-48), which declined to 27% (95% CI, -2-48) during days 61 to 126. In the cohort of all individuals aged ≥80 years, there were 5753 deaths during a median follow-up of 73 days and a maximum follow-up of 143 days. During days 7 to 60, the VE of the fourth dose was 71% (95% CI, 69-72), which declined to 54% (95% CI, 48-60) during days 61 to 143. The VE of the fourth dose seemed stronger when it was compared to third-dose recipients where at least four months had passed since vaccination (*P* < 0·001 for interaction).

**Interpretation:**

As compared with a third dose, a fourth dose of an mRNA COVID-19 vaccine, administered during the Omicron era, was associated with reduced risk of death from all causes in residents of LTCFs and in the oldest old during the first two months, after which the protection became slightly lower. These findings suggest that a fourth dose may prevent premature mortality in the oldest and frailest even after the emergence of the Omicron variant, although the timing of vaccination seems to be important with respect to the slight waning observed after two months.

**Funding:**

There was no funding source for this study.

**Research in context**

**Evidence before this study**

We searched PubMed and medRxiv without language restrictions for relevant literature until Apr 29, 2022, using key words such as “COVID-19”, “SARS-CoV-2”, “vaccine”, “booster”, “fourth dose”, “death”, “mortality”, “older adults”, and “long-term care facilities”. We found limited evidence on the effectiveness of a fourth dose of COVID-19 vaccine in old and frail people. Studies from Israel reported a high relative effectiveness from the fourth- compared to the third dose against COVID-19 related mortality in general older adults during one to two months. However, most of the participants were younger than 80 years and very few were residents of long-term care facilities (LTCFs), and the level of protection beyond the first two months remains incompletely understood. We also found no study having assessed all-cause mortality, which in the oldest and frailest people is of interest to study.

**Added value of this study**

This nationwide, retrospective, matched cohort study in Sweden showed that as compared with a third dose, a fourth dose of an mRNA COVID-19 vaccine, administered during a period when the Omicron variant of SARS-CoV-2 was dominating, was associated with substantially lower risk of death from all causes in LTCF residents and in people aged ≥80 years during the first two months. From thereon, the protection appeared to become slightly lower.

**Implications of all the available evidence**

As compared with the third dose, a second fourth dose of an mRNA COVID-19 vaccine, administered during the Omicron era, seems to cut the short-term risk of death from all causes in LTCF residents and in the oldest old. Given that the protection appeared to decline slightly after the first two months, the timing of vaccination seems to be important.

Alt-text: Unlabelled box

## Introduction

The emergence of the B.1.1.529 (Omicron) variant of SARS-CoV-2 changed the landscape of the pandemic drastically and generated a surge in new cases including among the oldest and frailest people living in long-term care facilities (LTCFs).^1^ Because these people have the highest risk of severe complications following an infection, precaution and protection of this population remains a public health priority. By 5 April 2022, a total of nine European countries including Sweden recommend a fourth dose of COVID-19 vaccine to certain vulnerable populations, such as residents in LTCFs and persons aged 80 years or older.^2^ Data on the protection afforded by the fourth dose in these people is urgently needed to inform vaccination policies and strategies and to prevent premature deaths.

Observational studies from Israel showed that in general older adults aged ≥60 years, a fourth dose of BNT162b2 was associated with lower rates of infection and severe illness^3^ and had about 75% effectiveness against COVID-19 mortality compared to the third dose, during an Omicron predominant period.^4^, ^5^, ^6^ However, all these studies were conducted in general older adults, where most were younger than 80 years and where LTCF residents were either excluded^4^ or represented only 3% of the sample.^6^ This means that their findings cannot be extrapolated to the most vulnerable people including the very old and those living in LTCFs, as these people may experience lower vaccine-induced protection while having a much higher risk of death.^2^^,^^7^ In addition, these previous studies covered only one to two months of follow-up, limiting the inferences with respect to durability of protection. To date, only one study from Canada has reported on the effectiveness of the fourth dose in a LTCF population, estimating a 40% relative effectiveness of a fourth dose of mRNA-1273 against COVID-19 hospitalisation or mortality.^8^ More evidence is clearly warranted. Importantly, because LTCF residents are rarely admitted to hospitals upon infection,^9^ and because the symptoms of severe infection may be absent, the effect of vaccination on total mortality is of interest to evaluate as it could capture undocumented COVID-19 mortality. Therefore, in this nationwide, registry-based, retrospective, matched cohort study, we investigated the relative effectiveness of a fourth dose of mRNA COVID-19 vaccine against all-cause mortality during up to 4.5 months of follow-up in LTCF residents and in the oldest old.

## Methods

### Study design and eligible populations

This was a retrospective, matched cohort study based on nationwide registry data. The study was approved by the Swedish Ethical Review Authority (no. 2021-00094) who waived the informed consent requirement given the retrospective design. Two study cohorts were formed. For the first cohort, individuals considered for inclusion were all LTCF residents with at least one registration in the Swedish national quality register Senior Alert during 2017-2020, and alive 1 January 2022 (*N* = 63,623). Senior Alert is a database of risk assessments performed in older adults aged ≥65 years focusing on assessment and prevention of falls, pressure ulcers, malnutrition, and oral health.^10^ The register captures an estimated 73% of all Swedish LTCF residents.^11^ For the second cohort, individuals considered for inclusion were all individuals living in Sweden and aged 80 years or older and alive 1 January 2022, obtained from the Total Population Register which is managed by Statistics Sweden.^12^ Information on vaccination status was obtained from the National Vaccination Register, which covers data on all vaccinations given to citizens in Sweden, including the date of dose/doses administration and the type of vaccine. Individuals with a previous documented SARS-CoV-2 infection were ineligible for inclusion. Data on previous infections were obtained from the SmiNet register. The National Vaccination Register and SmiNet register are both managed by the Public Health Agency of Sweden and healthcare providers are required to report to these registers according to Swedish law. During the follow-up period in the present study, individuals eligible for a fourth dose of an mRNA COVID-19 vaccine in Sweden were primarily people aged 80 years and over, people with homemaker service, and residents of LTCFs.^13^ The fourth dose was recommended to be administered at least four months after the receipt of the third dose.^13^ Starting from 28 April 2022, the recommendation was extended to those aged 65 years and over and to younger people with Down syndrome or immunodeficiency.^14^ The number of PCR-tests for SARS-CoV-2 and the incidence of SARS-CoV-2 during follow-up are shown in Supplemental Figure 1. The highest incidence of SARS-CoV-2 in Sweden observed during the time of follow-up in the present study was the all-time high during the pandemic. It should be noted, however, that in Sweden, the testing for the general public was terminated on 9 February 2022,^15^ which explains the rapid drop in confirmed infections from week 6 onwards. Yet, testing was still recommended to proceed in LTCFs.^15^

### Study cohorts

**Figure 1:**
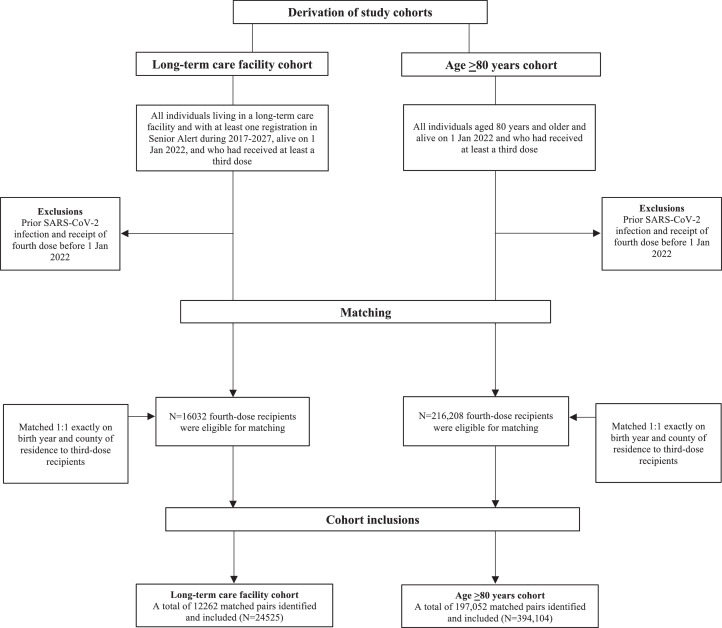
Selection and matching of participants for the two study cohorts.

Figure 1 presents the formation of study cohorts. For the first cohort, from the total population of eligible LTCF residents (*N* = 63,623), we selected all residents who had received at least the third dose (*N* = 45,160, referred to as third-dose recipients from hereon), and all individuals who had received at least the fourth dose (*N* = 22,630, referred to as fourth-dose recipients from hereon), and alive 1 January 2022. From the cohort of fourth-dose recipients, we excluded all individuals with a documented SARS-CoV-2 infection at the date of the fourth dose as in previous studies,^3^^,^^4^^,^^6^ and individuals given the fourth dose before 1 January, 2022, leaving 16,032 fourth-dose recipients eligible for matching. These individuals were matched 1:1 on birth year and county of residence in 2021, to all third-dose recipients. Baseline in both individuals of a matched pair was the date of the fourth dose in the fourth-dose recipient. The third-dose recipient in a matched pair was excluded if they at the assigned baseline date had either received the fourth dose, had died, or had a documented SARS-CoV-2 infection, whereby a new third-dose recipient was searched from the remainder of the cohort. This procedure was repeated four times resulting in a total study cohort of 12,262 matched pairs (*N* = 24,524). For the second cohort, we repeated the same matching procedure based on all individuals living in Sweden who were aged ≥80 years and who had received at least the third dose (*N* = 562,018). Individuals given the fourth dose from 1 January 2022 onwards (*N* = 216,208) were matched 1:1 on birth year and county of residence to all individuals given at least the third dose and alive on 1 January 2022, resulting in a matched cohort consisting of 197,052 pairs (*N* = 394,104).

### Outcome

The outcome of this study was all-cause mortality from 7 days after baseline until 27 May 2022 latest. Individual-level data on the exact date of death was collected from the Total Population Register, which is held by Statistics Sweden.^12^ In Sweden, the first documented case of the Omicron variant was reported on 29 November 2021,^16^ and by the beginning of 2022 it represented >90% of sequenced cases.^17^ Thus, as we began follow-up on 1 January 2022, the estimates of relative vaccine effectiveness in the present study are considered to be applicable to the Omicron era.

### Covariates

The covariates selected for the present study were based on results from previous studies based on similar populations.^9^^,^^18^ Information on sex, year and month of birth, and country of birth were obtained from Statistics Sweden. From Senior Alert, we obtained data on risk of malnutrition and risk of pressure ulcer assessed using validated instruments that was used in the description and analyses of the LTCF cohort.^10^ Malnutrition risk was assessed using the Mini Nutritional Assessment short-form (MNA-SF)^19^ and pressure ulcer risk was assessed using the Modified Norton Scale (MNS).^20^ Specifically, the MNA-SF assesses recent reductions in food intake, recent unintentional weight loss, mobility, recent psychological stress or acute disease, neuropsychological conditions, and body mass index.^19^ The MNS assesses current physical status, physical activity, movement ability, food intake, fluid intake, incontinence, and general condition.^20^

### Statistical analysis

Cumulative mortality was illustrated using the Kaplan-Meier method with estimated 95% confidence intervals (CI). To compare the risk of death based on vaccination status (fourth vs. third dose), Cox regression was used to calculate hazard ratios (HR). The 95% CI was estimated using robust standard errors to adjust for the matched cohorts. Follow-up time was censored at the date of additional vaccination (date of fifth dose in fourth-dose recipients and date of fourth dose in third-dose recipients), or date of death, or on 27 May 2022, whichever came first. To formally test whether the associations were time-dependent, Schoenfeld's residuals were evaluated using *estat phtest* command in Stata. Since the test indicated that the proportional hazard assumption was violated (*P* < 0·05) for the main exposure, the associations were investigated during days 7 to 60, and from 61 days onwards. In both cohorts, the first Cox model was adjusted for age and baseline date. In the LTCF cohort, the second model included the additional covariates sex (2 categories), whether the individual was born in Sweden (2 categories), MNS score (range 8-28 points), and MNA-SF score (range 0-14 points). In the cohort of all individuals aged ≥80 years, the second model included the additional covariates whether the individual was born in Sweden (2 categories), and whether the individual lived in own housing (two categories). The adjusted relative vaccine effectiveness (VE) of the fourth dose was calculated as (1–HR) × 100%. To investigate whether there was effect measure modification of the associations between the exposure and outcome by the covariates, interaction analyses were performed using product terms created by multiplying the variable coding for vaccination status at baseline by each respective covariate, which were added to the finally-adjusted Cox model. Moreover, we tested whether the dose interval modified the association by creating a product term between vaccination status and the number of days between vaccination with the third and fourth dose in fourth-dose recipients, and the number of days passed since the third dose and baseline date in third-dose recipients. Finally, we tested whether the type of vaccine given as fourth dose modified the association, by creating a product term between vaccination status and vaccine type given as fourth dose (BNT162b2 or mRNA-1273). All analyses were performed in SPSS v27·0 for Mac (IBM Corp, Armonk, NY, USA), and Stata v16·1 for Mac (Statcorp, College Station, Texas, USA). A two-sided P-value <0·05 or HR with 95% CIs not crossing one were considered statistically significant.

### Role of the funding source

There was no funding source for this study.

## Results

### Study cohorts

Baseline characteristics for the LTCF cohort are presented in Table 1. The median age was 86·4 years, about 68% were women, and almost 90% were born in Sweden. As shown, the individuals were in general frail, as the majority were diagnosed both with neuropsychological conditions such as dementia, and sometimes confusion. Fourth-dose recipients and third-dose recipients appeared to be well balanced at baseline. More than 98% of all participants had received BNT162b2 for primary-series vaccination. All third doses given were of an mRNA type, where about 80% were BNT162b2. Among fourth-dose recipients, about 60% received BNT162b2 and the remaining received mRNA-1273 as their fourth dose. During follow-up, 10,012 individuals (81·7%) in the third-dose group received a fourth dose, whereby they were censored.

**Table 1:** Baseline characteristics of the long-term care facility cohort.

	Fourth-dose group (*N* = 12,262)	Third-dose group (*N* = 12,262)
Baseline date, mean	4 March 2022	4 March 2022
Age, years	86·4 (80·1-91·8)	86·4 (80·1-91·8)
Female sex	8312 (67·8)	8340 (68·0)
Born in Sweden	10,947 (89·3)	10,878 (88·7)
Vaccination schedule		
BNT162b2 for primary-series	12,154 (99·1)	11,932 (97·3)
BNT162b2 as third dose	9915 (80·9)	9870 (80·5)
BNT162b2 as fourth dose	7197 (58·7)	
mRNA-1273 as fourth dose	5065 (41·3)	
Mean date of Senior Alert evaluation	15 April 2020	7 March 2020
MNA-SF score (0-14), mean (SD)	10·8 (2·4)	10·7 (2·5)
MNS score (8-28), mean (SD)	22·9 (3·3)	23·1 (3·3)
General condition		
Good	7032 (57·3)	7066 (57·6)
Pretty good	4318 (35·2)	4360 (35·6)
Bad	354 (2·9)	382 (3·1)
Very bad	25 (0·2)	22 (0·2)
Missing	533 (4·4)	432 (3·5)
Unintentional weight loss last three months		
No	8443 (68·9)	8234 (67·2)
1-3 kg	1785 (14·6)	1702 (13·9)
>3 kg	803 (6·5)	851 (6·9)
Unknown	1231 (10·0)	1475 (12·0)
Incontinence		
No	3970 (32·4)	4455 (36·3)
Temporary but unusual	1685 (13·7)	1608 (13·1)
Urinary but not bowel	2480 (20·2)	2576 (21·0)
Both urinary and bowel	3594 (29·3)	3191 (26·0)
Missing	533 (4·4)	432 (3·5)
Neuropsychological conditions		
None	3718 (30·3)	4340 (35·4)
Mild dementia or depression	5644 (46·0)	5391 (44·0)
Severe dementia or depression	2900 (23·7)	2531 (20·6)
Mental status		
Fully oriented	3595 (29·3)	4147 (33·8)
Sometimes disoriented	6860 (55·9)	6535 (53·3)
No proper response	1258 (10·3)	1138 (9·3)
Unreachable	16 (0·1)	100·1)
Missing	533 (4·4)	432 (3·5)
Movement ability		
Walking with or without aid	7238 (59·0)	7380 (60·2)
Walking with help of person	1369 (11·2)	1437 (11·7)
Dependent on wheelchair	2988 (24·4)	2869 (23·4)
Bedridden	134 (1·1)	144 (1·2)
Unknown	533 (4·4)	432 (3·5)

Data are median (IQR) or n (%) unless otherwise specified. IQR=interquartile range. MNA-SF=Mini Nutritional Assessment Short-Form. MNS=Modified Norton Scale. SD=standard deviation.

Baseline characteristics for the cohort of everyone aged ≥80 years are presented in Table 2. The median age was 85·2 years, about 58% were women, and about 7% were registered as living in a LTCF according to the Senior Alert register. Fourth-dose recipients were more often living in LTCFs compared to third-dose recipients (8·6% vs 5·4%). More than 80% of all participants had received BNT162b2 for primary-series vaccination. All third doses given were of an mRNA type, where 80% were BNT162b2. Among fourth-dose recipients, about 60% received BNT162b2 and the remaining received mRNA-1273. During follow-up, 157,981 individuals (80·2%) in the third-dose group received a fourth dose, whereby they were censored.

**Table 2:** Baseline characteristics of the cohort including all individuals aged 80 years and older.

	Fourth-dose group (*N* = 197,052)	Third-dose group (*N* = 197,052)
Baseline date, mean	6 March 2022	6 March 2022
Age, years	85·2 (82·6-88·7)	85·2 (82·6-88·7)
Female sex	113,781 (57·7)	116,521 (59·1)
Born in Sweden	179,690 (91·2)	176,013 (89·3)
Living in long-term care facility	17,003 (8·6)	10,738 (5·5)
Vaccination schedule		
BNT162b2 for primary-series	168,743 (85·6)	159,162 (80·8)
BNT162b2 as third dose	158,337 (80·4)	156,915 (79·6)
BNT162b2 as fourth dose	118,056 (59·9)	
mRNA-1273 as fourth dose	78,926 (40·1)	

Data are median (IQR) or n (%) unless otherwise specified. IQR = interquartile range.

### Effectiveness of the fourth dose in LTCF residents

**Figure 2:**
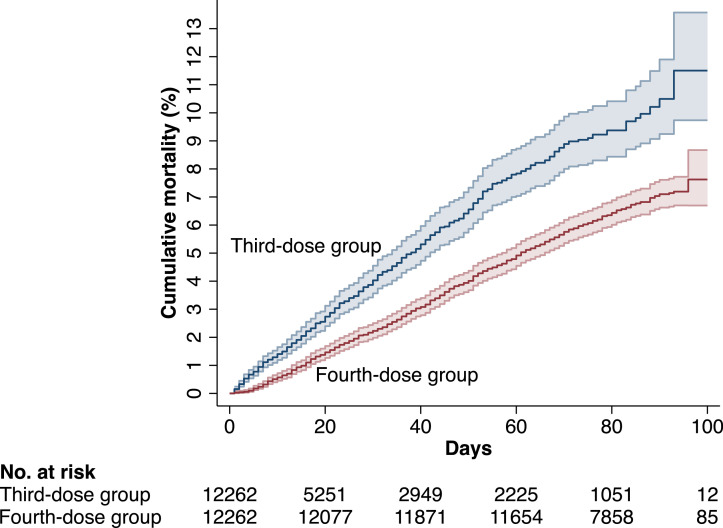
Cumulative risk of death in the fourth-dose group and the third-dose group during the first 100 days of follow-up in the cohort of long-term care facility residents.

The median (interquartile range [IQR]) follow-up time was 77 (25-86) days, with a maximum follow-up of 126 days, during which 1119 residents died. Figure 2 shows the cumulative risk of all-cause mortality during the first 100 days of follow-up. During days 7 to 60, the VE of the fourth dose against all-cause mortality was 39% (95% CI, 29-48, *P* < 0·001, Table 3), with no effect modification by the different covariates (P>0·05 for all interactions tested). There was also no significant effect modification by dose interval in fourth-dose recipients (P=0·60 for interaction), or by the time since first third dose and baseline in third-dose recipients (P=0·09 for interaction), or by type of vaccine given as fourth dose (P=0·50 for interaction). During days 61 to the maximum follow-up of 126 days, there were 259 deaths. During this period, the VE of the fourth dose was attenuated (VE, 27%, (95% CI, -2-48, P=0·07), Table 3).

**Table 3:** Relative vaccine effectiveness of the fourth dose against all-cause mortality in residents of long-term care facilities by number of days after the fourth dose, and according to sex, age, and time passed since vaccination.

	Fourth-dose group		Third-dose group		Relative vaccine effectiveness (95% CI)	
	Deaths	Deaths/100,000 person-days	Deaths	Deaths/100,000 person-days	Adjusted for age and baseline date	Fully adjusted*
7-60 days, total cohort (*N* = 21,623)	573	79·8	292	105·2	37 (27-45)	39 (29-48)
Men (*N* = 6996)	206	89·1	97	104·2	30 (11-45)	31 (12-46)
Women (*N* = 14,627)	367	75·4	195	105·7	40 (28-50)	43 (31-53)
Age >85 years (*N* = 12,030)	389	97·0	193	135·1	38 (26-48)	39 (27-49)
Age ≤85 years (*N* = 9593)	184	58·1	99	73·5	34 (16-49)	39 (21-52)
>4 months since vaccination in the third-dose group (*N* = 16,809)	470	82·2	223	122·0	45 (35-53)	46 (36-55)
≤4 months since vaccination dose in the third-dose group (*N* = 4814)	107	70·6	69	72·8	15 (-16-38)	20 (-10-43)
>4 months since vaccination in the fourth-dose group (*N* = 20,927)	549	79·0	287	106·9	38 (29-47)	41 (31-49)
61-126 days, total remaining cohort (*N* = 13,853)	214	22·0	45	25·5	30 (3-49)	27 (-2-48)

CI=confidence interval.

⁎ Adjusted for age, baseline date, sex, born in Sweden, score on Modified Norton Scale, and score on Mini Nutritional Assessment Short-Form Scale.

### Effectiveness of the fourth dose in individuals aged 80 years and older

**Figure 3:**
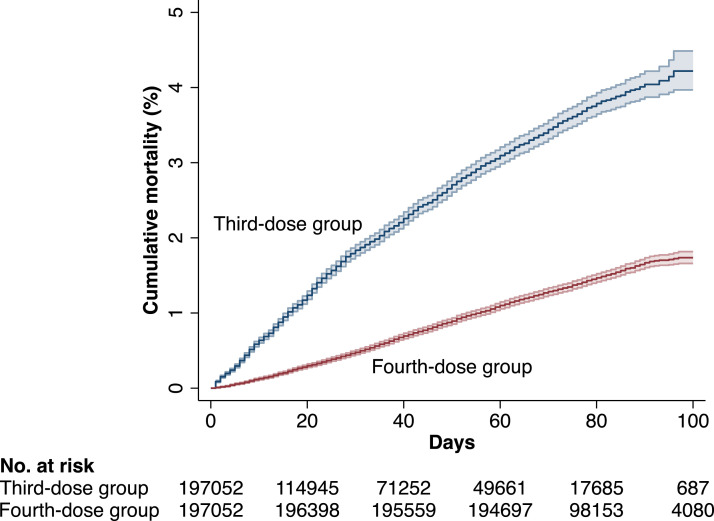
Cumulative risk of death in the fourth-dose group and the third-dose group during the first 100 days of follow-up in the cohort including all individuals aged 80 years and older.

The median (IQR) follow-up time was 73 (35-84) days, with a maximum follow-up of 143 days, during which 5753 individuals died. Figure 3 shows the cumulative risk of all-cause mortality during the first 100 days of follow-up. During days 7 to 60, the VE of the fourth dose against all-cause mortality was 71% (95% CI, 69-72, *P* < 0·001, Table 4). There was a significant effect modification by living conditions (*P* < 0·01 for interaction), with a slightly higher VE for individuals living in their own home (VE, 73%, (95% CI, 71-75, *P* < 0·001). We also found evidence of effect modification related to the time passed since vaccination in third-dose recipients (*P* < 0·001 for interaction), where if including only third-dose recipients where ≥4 months had passed since vaccination, the VE of the fourth dose increased to 79% (95% CI, 77-81, *P* < 0·001). In contrast, there was no significant effect modification by time between the third and fourth dose, in fourth-dose recipients (P=0·10). During days 61 up to the maximum follow-up of 143 days, there were 1054 deaths. During this period, the VE of the fourth dose was attenuated to 54% (95% CI, 48-60, *P* < 0·001, Table 4).

**Table 4:** Relative vaccine effectiveness of the fourth dose against all-cause mortality in individuals aged 80 years and older by number of days after the fourth dose, and according to sex, age, and time passed since vaccination.

	Fourth-dose group		Third-dose group		Relative vaccine effectiveness (95% CI)	
	Deaths	Deaths/100,000 person-days	Deaths	Deaths/100,000 person-days	Adjusted for age and baseline date	Fully adjusted*
7-60 days, total cohort (*N* = 365,249)	2040	17·4	2659	45·3	64 (62-66)	71 (69-72)
Men (*N* = 151,823)	887	17·9	1195	50·5	67 (65-70)	71 (69-74)
Women (*N* = 213,426)	1153	17·0	1464	41·8	62 (59-65)	70 (68-72)
Age >85 years (*N* = 186,451)	1520	25·2	1957	66·4	64 (62-67)	71 (69-73)
Age ≤85 years (*N* = 178,798)	520	9·1	702	24·0	65 (61-69)	70 (67-74)
>4 months since vaccination in the third-dose group (*N* = 134,831)	859	18·5	1353	80·6	78 (77-80)	79 (77-81)
≤4 months since vaccination in the third-dose group (*N* = 230,418)	1181	16·6	1,306	31·2	50 (46-54)	61 (58-64)
>4 months since vaccination in the fourth-dose group (*N* = 308,446)	1815	18·3	2298	46·3	63 (61-66)	71 (69-72)
61-143 days, total remaining cohort (*N* = 243,880)	757	4·8	297	7·8	46 (38-53)	54 (48-60)

CI=confidence interval.

⁎ Adjusted for age, baseline date, sex, born in Sweden, and residence in long-term care facility.

## Discussion

The results of this study suggest that as compared with a third dose, a fourth dose of an mRNA COVID-19 vaccine cut the short-term risk of death from all causes by about 40% in LTCF residents, and by more than 70% in the oldest old living at home during a period when the Omicron variant of SARS-CoV-2 was dominating. After two months, the protection appeared to begin declining slightly. Our findings indicate that promoting a high uptake of the fourth dose may help prevent premature mortality in frail older individuals, and that timely administration of the doses is important.

Evidence on the effectiveness of the fourth dose in the most vulnerable part of the population is sparse. Israeli studies have estimated more than 70% effectiveness against COVID-19 mortality in individuals aged 60 years and older during one to two months of follow-up.^4^, ^5^, ^6^ However, 75% to 80% of those participants were below 80 years of age, thereby limiting the possibility to make inferences on the protection afforded by the fourth dose in the oldest and frailest. Also, none of the studies assessed all-cause mortality. In the present study, we found a similar estimate of relative vaccine effectiveness against the outcome of all-cause mortality during the first two months in a nationwide cohort of individuals aged 80 years and over, especially among those living in their own home. We also found that the relative protection afforded by the fourth dose increased when it was compared to third-dose recipients who had been vaccinated more than 4 months ago, which indicates a rather limited durability of protection from the third dose in our cohort. Importantly, whereas previous studies have provided estimates of protection limited to the first two months after the fourth dose, we also studied the protection beyond this period. As shown, the protection from the fourth dose seemed to begin waning slightly already after the first two months. Taken together, our findings have important implications as they emphasize timely administration of the fourth dose in old and frail individuals. This is supported by findings from previous studies showing that the effect of COVID-19 vaccination is waning with older age, and especially after the age of 80.^7^^,^^21^

However, age per se does not fully capture the heterogeneity of older individuals, as excess mortality during the pandemic have been many times higher in LTCF residents compared to those of the same age who lives alone,^22^ probably because residents in LTCFs are frailer and because transmission in LTCFs have been high. This makes it critical to obtain evidence on the effects of vaccination in this population specifically. We found that the fourth dose reduced all-cause mortality by about 40% during the first two months in LTCF residents. No previous studies are available for direct comparison with these results, but one study from Canada reported a 40% reduced risk of COVID-19 hospitalisation or death from the fourth- compared to the third dose in LTCF residents.^8^ The outcome in that study was however restricted to hospitalisations or deaths following a positive PCR-test, which may increase the risk of misclassification, especially in this population. Irrespectively, the results from our study indicate benefits of the fourth dose on severe outcomes in this population consisting of the frailest old individuals. Importantly, also in this cohort we found that the protection became attenuated after more than two months.

The present study has limitations that should be considered. Despite the matching and adjustment for various covariates, the associations may be susceptible to residual and unmeasured confounding. For example, we did not have access to data on various covariates that may influence the risk of death, especially in the second cohort, although in a previous nationwide study on vaccine effectiveness where this data was available, the estimates were only marginally affected by adjustment for eight comorbidities.^21^ However, access to such data would still have been of interest to allow us to also explore whether the effectiveness of the fourth dose differed across subgroups.

Furthermore, although third-dose recipients had similar baseline characteristics as fourth-dose recipients, some third-dose recipients likely did not receive the fourth dose because of deteriorating health that was not captured by the baseline characteristics. If so, this would increase their risk of death and result in a higher estimated VE. Finally, although it was not our primary aim, we were unable to estimate the vaccine effectiveness specifically against COVID-19 related death because data on cause-specific mortality is not available from the Total Population Register. Given that COVID-19 mortality is the outcome used in all been more widely assessed in current studies, an assessment of this outcome also in the present study may have facilitated comparisons with both previous and ongoing/future studies. There are also strengths, of which a key one is the nationwide cohorts evaluated and the access to several covariates in the LTCF cohort that captured physical condition and frailty, altogether increasing the internal- and external validity of the findings. Our follow-up time was also longer than previous studies evaluating the protection from the fourth dose, thereby allowing us to study the level of protection beyond the first two months. Another potential strength is the assessment of all-cause mortality, which likely produced more reliable and less biased estimates for this population as opposed to outcomes such as COVID-19 hospitalisation or death after a positive PCR-test, given that the oldest and frailest individuals living in LTCFs are rarely admitted to hospitals,^9^ and because symptoms of a severe infection may be absent. However, it should be noted that the outcome of all-cause mortality will also include deaths from other causes than COVID-19, with lower resulting estimates than for the outcome of COVID-19 related death.

In conclusion, this nationwide study suggests that a fourth dose of an mRNA COVID-19 vaccine reduces premature mortality from all causes among residents in LTCFs and in the oldest old, as compared with a third dose. Accordingly, promoting a high uptake of the fourth dose in the oldest and frailest people may help prevent premature deaths, even after the emergence of the Omicron variant for which disease severity appears reduced as compared to the earlier variants.^23^ Yet, the slight waning that became evident after two months suggests that timely administration of these doses is important.

## Contributors

P.N. and M.B. conceived and designed the study. P.N. acquired the data. P.N. constructed the data files for analysis and performed the matching. P.N. performed the statistical analyses, supported by M.B., P.N. and M.B. accessed and verified the underlying data. All authors interpreted the data. M.B. drafted the manuscript, supported by P.N. All authors critically revised the manuscript for intellectual content. P.N. and A.N. supervised the work. All authors gave final approval of the version to be published. All authors had full access to all the data and had final responsibility for the decision to submit for publication.

## Data sharing statement

The data files used for the present study is publicly unavailable according to regulations under Swedish law. However, all data used for the present study can be applied for from the National Board of Health and Welfare, Statistics Sweden, and the Public Health Agency of Sweden.

## Declaration of interests

We declare no competing interests.

## Appendix Supplementary materials

Image, application 1

## References

[1] Public Health Agency of Sweden. Basis for Decision on the Recommendation of a Second Booster Dose (dose 4) of Covid-19 Vaccine, 2022. https://www.folkhalsomyndigheten.se/publicerat-material/publikationsarkiv/b/beslutsunderlag-om-rekommendation-av-en-andra-pafyllnadsdos-dos-4-av-covid-19-vaccin/. Accessed 1 April 2022.

[2] European Centre for Disease Prevention and Control. COVID-19: Joint statement from ECDC and EMA on the administration of a fourth dose of mRNA vaccines, 2022. https://www.ecdc.europa.eu/en/news-events/ema-ecdc-statement-fourth-covid-vaccine-dose. Accessed 8 April 2022.

[3] Bar-On YM, Goldberg Y, Mandel M (2022). Protection by a Fourth Dose of BNT162b2 against Omicron in Israel. N Engl J Med.

[4] Magen O, Waxman JG, Makov-Assif M (2022). Fourth dose of BNT162b2 mRNA Covid-19 vaccine in a nationwide setting. N Engl J Med.

[5] Arbel R, Sergienko R, Friger M (2022). Effectiveness of a second BNT162b2 booster vaccine against hospitalization and death from COVID-19 in adults aged over 60 years. Nat Med.

[6] Gazit S, Saciuk Y, Perez G, Peretz A, Pitzer VE, Patalon T. (2022). Short term, relative effectiveness of four doses versus three doses of BNT162b2 vaccine in people aged 60 years and older in Israel: retrospective, test negative, case-control study. BMJ.

[7] Arregocés-Castillo L, Fernández-Niño J, Rojas-Botero M (2022). Effectiveness of COVID-19 vaccines in older adults in Colombia: a retrospective, population-based study of the ESPERANZA cohort. Lancet Healthy Longev.

[8] Grewal R, Kitchen SA, Nguyen L (2022). Effectiveness of a fourth dose of covid-19 mRNA vaccine against the omicron variant among long term care residents in Ontario, Canada: test negative design study. BMJ.

[9] Bergman J, Ballin M, Nordstrom A, Nordstrom P. (2021). Risk factors for COVID-19 diagnosis, hospitalization, and subsequent all-cause mortality in Sweden: a nationwide study. Eur J Epidemiol.

[10] Edvinsson J, Rahm M, Trinks A, Hoglund PJ. (2015). Senior alert: a quality registry to support a standardized, structured, and systematic preventive care process for older adults. Qual Manag Health Care.

[11] (2019). https://plus.rjl.se/info_files/infosida40605/118_Senior_alert_Arsrapport_2019.pdf.

[12] Ludvigsson JF, Almqvist C, Bonamy A-KE (2016). Registers of the Swedish total population and their use in medical research. Eur J Epidemiol.

[13] Public Health Agency of Sweden. The second booster dose of covid-19 vaccine is recommended for people aged 80 and over, 2022. https://www.folkhalsomyndigheten.se/nyheter-och-press/nyhetsarkiv/2022/februari/andra-pafyllnadsdos-vaccin-mot-covid-19-rekommenderas-till-personer-som-ar-80-ar-och-aldre/. Accessed 29 March 2022.

[14] Public Health Agency of Sweden. Decision basis for extended recommendation for a second booster dose of covid-19 vaccine - For all persons 65 years and older, as well as persons 18–64 years with Down syndrome and persons 18–64 years with moderate to severe immunodeficiency. https://www.folkhalsomyndigheten.se/publicerat-material/publikationsarkiv/b/beslutsunderlag-for-utvidgad-rekommendation-om-en-andra-pafyllnadsdos-covid-19-vaccin/. Accessed 10 June 2022.

[15] Public Health Agency of Sweden. Most actions against covid-19 ends on February 9, 2022. https://www.folkhalsomyndigheten.se/nyheter-och-press/nyhetsarkiv/2022/februari/de-flesta-atgarder-mot-covid-19-upphor-den-9-februari/. Accessed 1 April 2022.

[16] Public Health Agency of Sweden. New virus variant of SARS-CoV-2 also in Sweden, 2021. https://www.folkhalsomyndigheten.se/nyheter-och-press/nyhetsarkiv/2021/november/ny-virusvariant-av-sars-cov-2-aven-i-sverige/. Accessed 29 March 2022.

[17] Public Health Agency of Sweden. Statistics on SARS-CoV-2 virus variants of particular importance, 2022. https://www.folkhalsomyndigheten.se/smittskydd-beredskap/utbrott/aktuella-utbrott/covid-19/statistik-och-analyser/sars-cov-2-virusvarianter-av-sarskild-betydelse/. Accessed 29 March 2022.

[18] Ballin M, Bergman J, Kivipelto M, Nordström A, Nordström P. (2021). Excess mortality after COVID-19 in Swedish long-term care facilities. J Am Med Dir Assoc.

[19] Kaiser MJ, Bauer JM, Ramsch C (2009). Validation of the Mini Nutritional Assessment short-form (MNA-SF): a practical tool for identification of nutritional status. J Nutr Health Aging.

[20] Pancorbo-Hidalgo PL, Garcia-Fernandez FP, Lopez-Medina IM, Alvarez-Nieto C. (2006). Risk assessment scales for pressure ulcer prevention: a systematic review. J Adv Nurs.

[21] Nordström P, Ballin M, Nordström A. (2022). Risk of infection, hospitalisation, and death up to 9 months after a second dose of COVID-19 vaccine: a retrospective, total population cohort study in Sweden. Lancet North Am Ed.

[22] Modig K, Lambe M, Ahlbom A, Ebeling M. (2021). Excess mortality for men and women above age 70 according to level of care during the first wave of COVID-19 pandemic in Sweden: A population-based study. Lancet Reg Health Eur.

[23] Krutikov M, Stirrup O, Nacer-Laidi H (2022). Outcomes of SARS-CoV-2 omicron infection in residents of long-term care facilities in England (VIVALDI): a prospective, cohort study. Lancet Healthy Longev.

