# Supplementary material for: Effectiveness of a fourth dose of mRNA COVID-19 vaccine against all-cause mortality in long-term care facility residents and in the oldest old: A nationwide, retrospective cohort study in Sweden
Source: Lancet Reg Health Eur. 2022 Jul 13;21:100466. doi: 10.1016/j.lanepe.2022.100466 (PMC9277096; doi:10.1016/j.lanepe.2022.100466)

Supplementary Appendix to:

Effectiveness of a fourth dose of mRNA COVID-19 vaccine against all-cause mortality in long-term care facility residents and in the oldest old: a nationwide, retrospective cohort study in Sweden

Prof. Peter Nordström, PhD*, Marcel Ballin, MSc*, Anna Nordström, PhD

*Contributed equally as joint first authors.

Affiliations
Department of Community Medicine and Rehabilitation, Unit of Geriatric Medicine, Umeå University, Umeå, Sweden (Peter Nordström, Marcel Ballin and Anna Nordström)
Department of Public Health and Clinical Medicine, Section of Sustainable Health, Umeå University, Umeå, Sweden (Anna Nordström)
School of Sport Sciences, UiT the Arctic University of Norway, Tromsø, Norway (Anna Nordström)

Contents

Supplemental Figure 1. The number of PCR-tests performed (A) and the incidence of confirmed SARS-CoV-2 infections (B) in the total population of Sweden during the follow-up period in the present study. The data was derived from publicly available data at Public Health Agency of Sweden. 2

Supplemental Figure 1. The number of PCR-tests performed (A) and the incidence of confirmed SARS-CoV-2 infections (B) in the total population of Sweden during the follow-up period in the present study. The data was derived from publicly available data at Public Health Agency of Sweden.

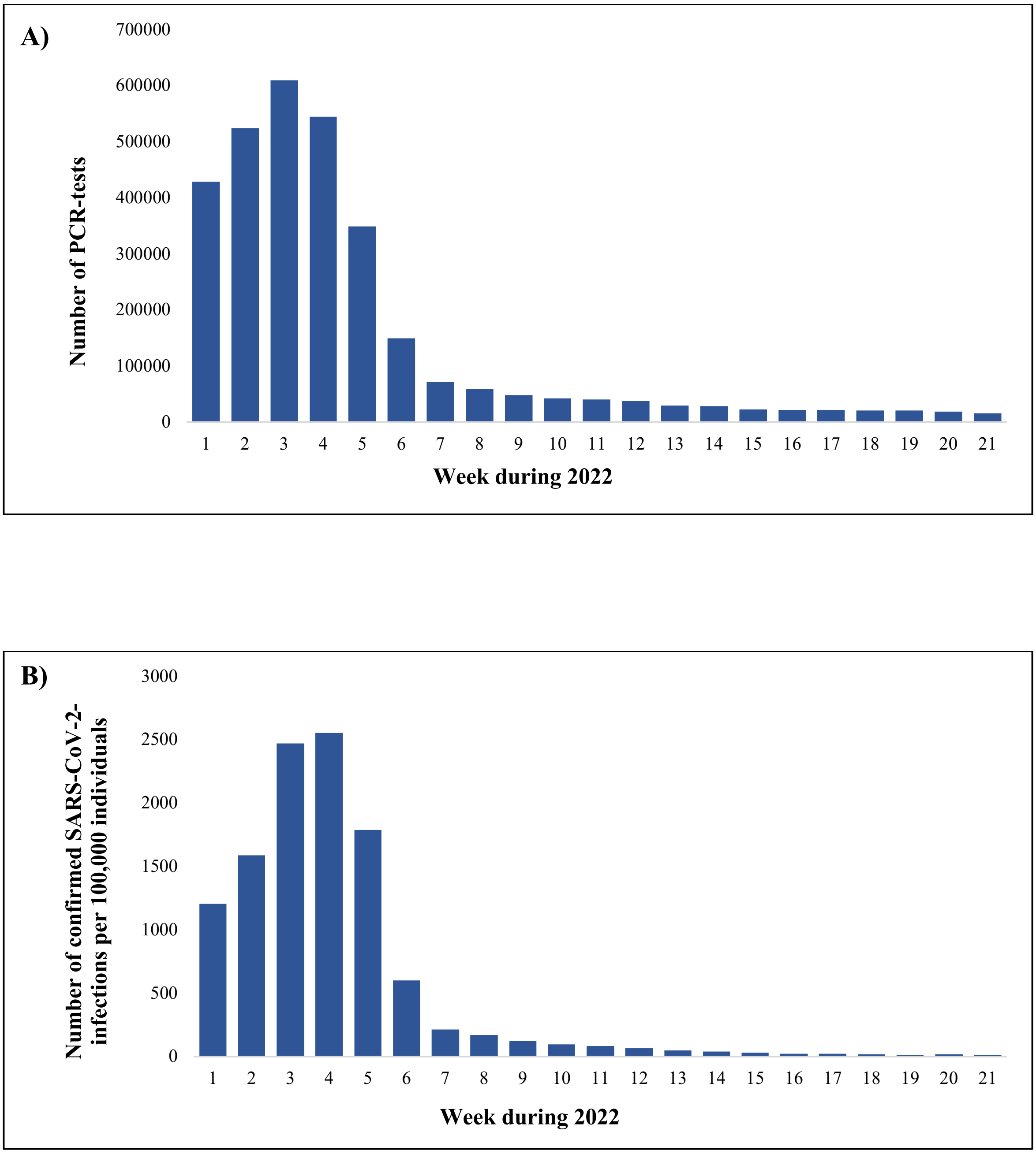

Supplement: Supplementary file 1 [file mmc1.pdf]
